# A comparative analysis of volatile organic compounds in Paeoniae Radix Alba processed using different methods

**DOI:** 10.3389/fnut.2025.1675150

**Published:** 2025-10-24

**Authors:** Yuqing Liu, Yafang Du, Yadan Xiao, Yue Wu, Dan Huang, Xuan Guo

**Affiliations:** ^1^School of Stomatology, Hunan University of Chinese Medicine, Changsha, China; ^2^State Key Laboratory of Chinese Medicine Powder and Medicine Innovation in Hunan (Incubation), Science and Technology Innovation Center, Hunan University of Chinese Medicine, Changsha, China; ^3^School of Pharmacy, Hunan University of Chinese Medicine, Changsha, China

**Keywords:** Paeoniae Radix Alba, volatile organic compounds, processing methods, GC-IMS, Heracles NEO ultra-fast gas phase electronic nose

## Abstract

**Introduction:**

Paeoniae Radix Alba (PRA) is a popular functional food, and volatile organic compounds (VOCs) are a critical aspect of PRA. Different processing methods often have a certain impact on VOCs.

**Methods:**

To investigate which VOCs have changed due to different processing methods, this study utilized gas chromatography–ion mobility spectrometry (GC-IMS), the Heracles NEO ultra-fast gas phase electronic nose (E-nose), and chemometric methods to compare and analyze the VOCs in PRA subjected to different processing methods: PRA (prepared slices, stir-baked, stir-baked with bran and stir-baked with wine).

**Results:**

A total of 85 VOCs were detected. Chemometric methods such as principal component analysis (PCA), cluster analysis (CA), and partial least squares regression analysis (PLS-DA) revealed that different processing methods have a significant impact on the VOCs of PRA. Among the PRA samples subjected to the three processing methods, the difference between PRA (prepared slices) and PRA (stir-baked with wine) is the largest. A higher amount of linalool oxide, is present in PRA (prepared slices) than in the other groups. The content of propanoic acid is highest in PRA (stir-baked with wine).

**Discussion:**

The differences in VOCs between PRA (stir-baked) and PRA (stir-baked with bran) are small. This study provides a theoretical basis for the development and clinical scientific application of functional foods related to PRA in the future.

## 1 Introduction

Paeoniae Radix Alba (PRA) is not only a well-known health-promoting food and traditional Chinese medicine (TCM), but also has a long history and wide applications in TCM clinical PRA ctice. It is a part of Shaoyao Gancao Decoction, Angelica and Peony Powder, and Free Wanderer Powder (1, 2), for example. Functional foods prepared using PRA include those suitable for patients with chemical liver injury—Baishao Danggui Wuweizi capsules and Gegen Fengjiao Baishao Zhizi capsules—and those with a laxative function: Danggui Baishao Luhui Tonifying capsules. It is commonly used to treat a variety of diseases, including cardiovascular disease, menstrual disorders, abdominal pain, diarrhea, liver disease, tumors, and rheumatoid arthritis (3–6). Its main active ingredients are paeoniflorin (7–9) and volatile organic compounds (VOCs) (10, 11).

Different processing methods have varying effects on the pharmacological components and activities of Paeoniae Radix Alba; For example, the analgesic effects of Paeoniae Radix Alba (stir-baked) and other processed products are stronger than those of PRA (prepared slices) (12). After PRA is stir-baked with bran, the contents of compounds with paeoniflorin structures and compounds similar to paeoniflorin, such as alloying glycosides, significantly increase (13). PRA (stir-baked with wine) can nourish the blood and promote blood circulation (14). Through processing, the proportion of paeoniflorin in PRA has decreased, while the proportion of albiflorin has increased (15). Despite various studies have been conducted on PRA and the various processing methods used in herb, there are almost no studies have compared and analyzed the VOCs of PRA processed using different methods. This gap in knowledge hinders a deeper understanding of how processing affects the chemical composition and therapeutic properties of PRA.

Volatile organic compounds are a crucial class of medicinal ingredients in PRA, but they have poor stability and are prone to volatilization and oxidation during processing. The commonly used methods for detecting VOCs in modern research include electronic noses (16), electronic tongues (17), Gas chromatography-Mass Spectrometry (GC-MS) (18), and Gas chromatography–ion mobility spectrometry (GC-IMS) (19). An E-nose is a device designed to identify and classify odors. The device is built around a series of sensors that detect the presence of odors, especially VOCs. And it generates electrical signals (voltages) called electronic nose data that contain chemical information. In recent years, E-noses have been widely used in food studies (20, 21). GC-IMS is an emerging technique used for the sensitive and selective detection of VOCs. Spectra obtained via GC-IMS are used as “fingerprints” for multivariate chemometric data analysis to extract information (22). Currently, the use of a single analytical technique may not be comprehensive enough to detect some important compounds. As such, the integration of multiple analytical techniques has become a popular research trend, offering more comprehensive, reliable, and scientific information (23).

This study aims to compare the VOCs of PRA (prepared slices) and its processed products, mainly investigating the differences in the VOCs of PRA (prepared slices), PRA (stir-baked), PRA (stir-baked with bran), and PRA (stir-baked with wine). The Heracles NEO ultra-fast gas phase electronic nose and GC-IMS were utilized to analyze the effects of different processing methods on the VOCs of PRA, and statistical analysis was conducted using principal component analysis (PCA), cluster analysis (CA), and partial least squares regression analysis (PLS-DA). This paper provides theoretical support for the scientific application of different PRA processed products in clinical PRA ctice and the development of functional foods in the future.

## 2 Materials and methods

### 2.1 Materials

Paeoniae Radix Alba is the dried root of *Paeonia lactiflora* Pall. It is collected in autumn, in 2024 (The samples were identified by Mr Zhaoming Xie at the Hunan Academy of Traditional Chinese Medicine, China), washed clean, removed from two ends and rootlet, and dried in the sun. They were collected from Anhui, China.

### 2.2 Sample preparation

Paeoniae Radix Alba (prepared slices): Weigh 100 g of PRA (prepared slices) as a single batch; crush to powder at once. Named BS-01.

Paeoniae Radix Alba (stir-baked) (150-160°C): Weigh 100 g of PRA (prepared slices) as a single batch and place this in a stir-baking container. Heat over low heat until slightly yellow, remove, cool, and then crush to powder at once. Named BS-02.

Paeoniae Radix Alba (stir-baked with bran) (150-160°C): First, heat the stir-baking container until 10 g of bran is lightly toasted and emits smoke. Next, add 100 g of PRA (prepared slices) as a single batch and stir-bake quickly until the surface turns yellow or dark yellow. Remove, sieve off the bran, let the sample cool, and crush to powder at once. Named BS-03.

Paeoniae Radix Alba (stir-baked with wine) (150-160°C): Take 100 g of PRA as a single batch, add 10 g of yellow wine, mix well, cover completely, place in a stir-baking container, stir-bake over low heat until slightly yellow, remove, cool, and then crush to powder at once. Named BS-04.

### 2.3 GC-IMS analysis

The VOCs in the dried powders were analyzed directly via GC-IMS using a FlavorSpec^®^ Gas Phase Ion Mobility Spectrometer from GAS (Dortmund, Germany).

Six ketones (2-butanone, 2-pentanone, 2-hexanone, 2-heptanone, 2-octanone, and 2-non-anone) were detected, and a calibration curve of retention time and retention index was established. Each sample (1 g) was placed in a 20 mL headspace vial and incubated for 15 min at 70°C. After that, 300 μL was injected into the headspace via non-shunt injection, and the vials were rotated at 500 rpm for 20 min (injection needle temperature: 85°C). An MXT-WAX capillary column (15 m × 0.53 mm, 1.0 μm, Restek Inc., Edmond, OK, USA) was used to separate the VOCs. The initial flow rate was 2.00 mL/min; this was increased linearly to 10.00 mL/min within 8 min and then to 100.00 mL/min within 10 min, where it was held for 30 min. The total runtime was 50 min, and the injection temperature was 85°C. Each sample was measured in three parallel groups.

The IMS conditions were as follows: IMS reagent: 3H (tritium); electric field intensity: 500 V/cm; drift tube temperature: 45°C; carrier gas: high-purity N2 (99.999%); flow rate: 75 mL/min; positive ion mode.

### 2.4 Heracles NEO ultra-fast gas phase electronic nose analysis

The sample was analyzed using a Heracles NEO ultra-fast gas chromatography electronic nose (Alpha MOS Corporation, Toulouse, France) with the following parameters: a sample weight of 2.0 g, incubation temperature of 80°C, and incubation time of 20 min. The initial trap temperature was 30°C, which was increased to a final temperature of 240°C. The flow rate of the trap was 10 mL/min, and the capture duration was 65 s. The inlet temperature was 200°C, the injection volume was 5000 μL, the speed was 250 μL/s, and the duration was 60 s. The initial column temperature was 40°C, which was increased at a rate of 0.7°C/s to 200°C and then at a rate of 3°C/s to 250°C. The acquisition time was 280 s, and the detector temperature was 260°C. AlphaSoft 2023 software (Alpha MOS Corporation, Toulouse, France) was used for data processing. Standard solutions of n-alkanes (nC6-nC16) were used for calibration, and the retention time was converted into a retention index. The VOCs were analyzed qualitatively using the AroChemBase database (Alpha MOS Corporation, Toulouse, France).

### 2.5 Statistical analysis

Reporter and Gallery Plot were utilized in VOCal data processing software (G.A.S., Dortmund, Germany, version 2.0.0) to analyze the VOCs. PCA was carried out using OmicShare Tools, while PLS-DA was performed using TBtools and SIMCA (Version 14.1, Umetrics, Sweden).

## 3 Results

### 3.1 The influence of different processing methods on the appearance of Paeoniae Radix Alba

**FIGURE 1:**
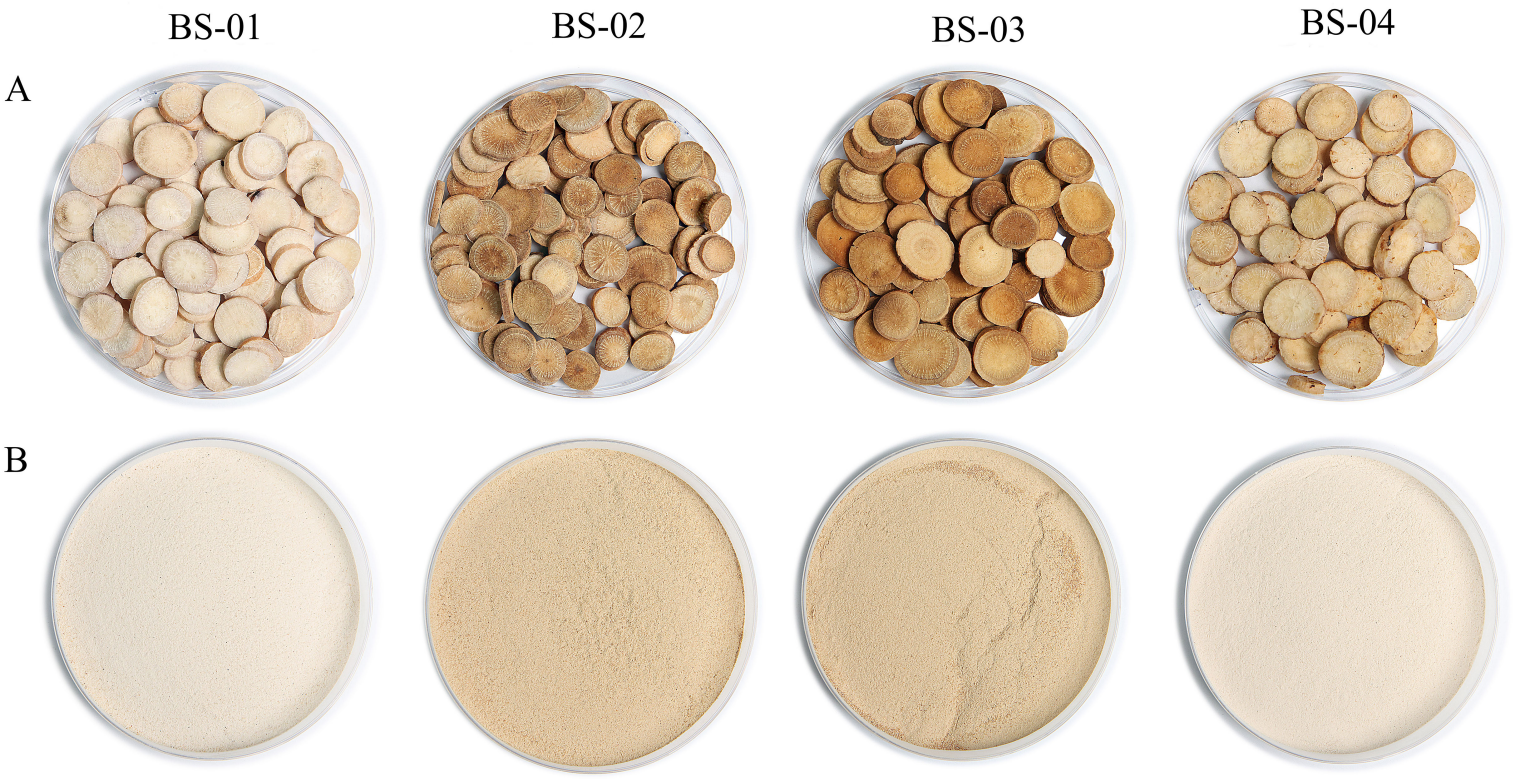
**(A)** Photos of PRA processed using different methods. **(B)** Photos of PRA powders processed using different methods.

The appearance of PRA before and after undergoing different processing methods is shown in Figure 1. The three processing methods left the original shape (circular) intact, but the color differed significantly. BS-04 (stir-baked with wine) had a yellow color, while BS-02 (stir-baked) and BS-03 (stir-baked with bran) had a burnt yellow color.

### 3.2 GC-IMS analysis results

#### 3.2.1 Compare differences in VOCs

**FIGURE 2:**
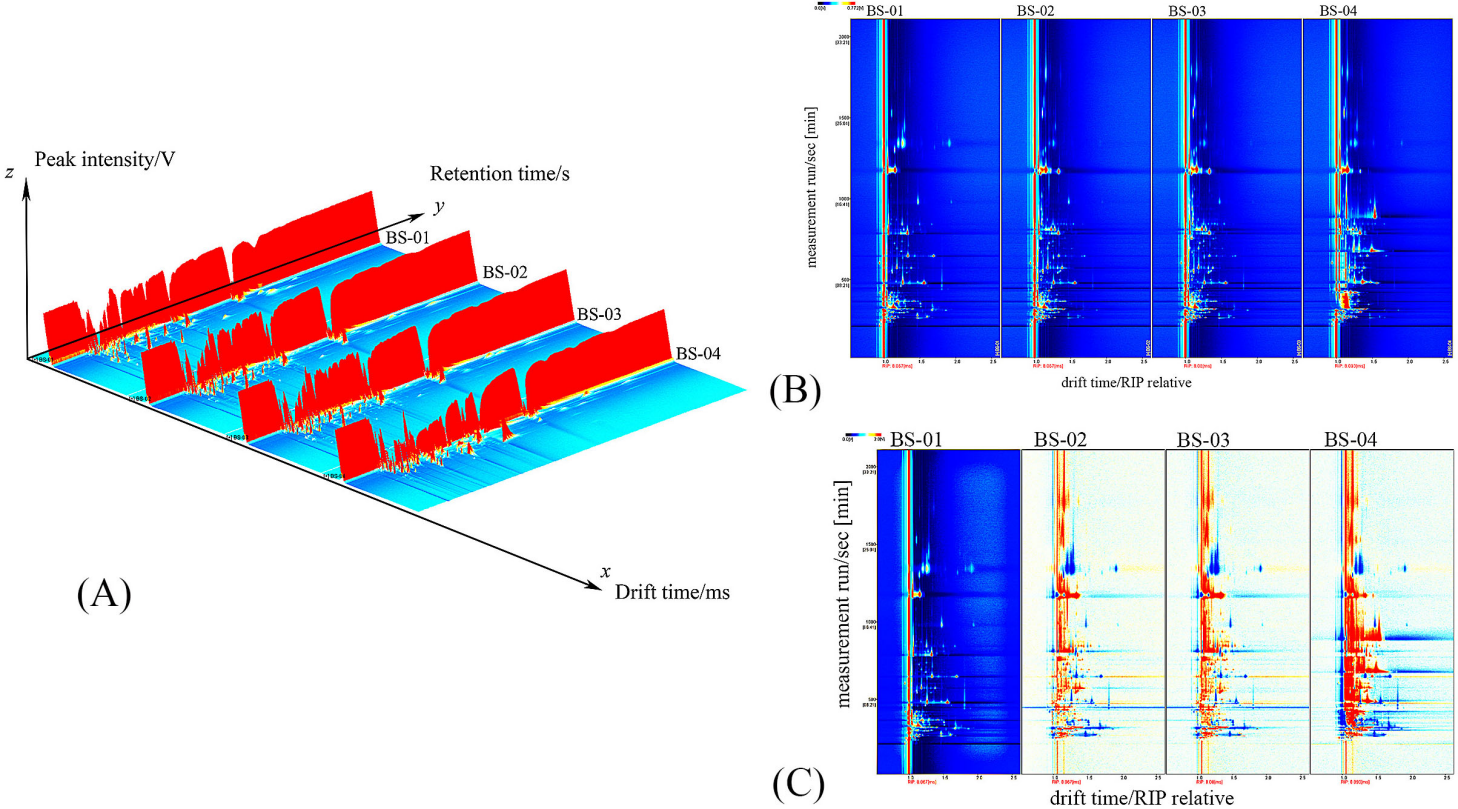
**(A)** Three-dimensional spectra of VOCs in four samples; **(B)** two-dimensional spectra of VOCs in four samples; **(C)** spectral comparison of PRA (prepared slices) with the other three groups.

The three coordinate axes in Figure 2A represent migration time (*X*-axis), retention time (*Y*-axis), and signal peak intensity (*Z*-axis), respectively. And Figure 2A shows the 3D GC-IMS spectrum, where the differences in VOCs between different samples can be observed. For ease of observation, convert to Figure 2B for display.

Figure 2B presents a top-down view, with blue representing the background and a vertical red line indicating the RIP peak (normalized reaction ion peak) at position 1.0 on the horizontal axis. The *y*-axis represents the retention time (s) under gas chromatography, while the *x*-axis denotes the relative migration time (a.u., normalized). The points on either side of the RIP peak represent individual VOCs, with the color indicating the peak intensity, ranging from blue to red, and darker colors indicating stronger peaks.

Figure 2B depicts differences in the VOCs of the PRA samples. To facilitate the comparison, the spectrum of the BS-01 sample was chosen as a reference. Spectra of other samples, with the reference spectrum subtracted, were used to create a comparison chart of differences, as shown in Figure 2C. If the volatile organic compound content in the target sample is identical to that in the reference, the subtracted background will be white. Red indicates a higher substance concentration in the target sample than the reference, while blue indicates a lower substance concentration.

#### 3.2.2 Qualitative analysis of volatile component spectra in four samples

A total of 60 VOCs were detected in the four samples, i.e., 20 aldehydes, 14 alcohols, 12 ketones, 6 esters, 3 organic acids, 3 heterocycles, and 2 sulfides. The detailed list of VOCs is provided in Table 1.

**TABLE 1:** Results of component analysis of VOCs in four samples.

NO	Classification	Compound	CAS	Molecular formula	MW	RI	Rt [sec]	Dt [a.u.]
1	Acids	Acetic acid M	64-19-7	C2H4O2	60.1	1447.6	1174.707	1.15662
2	Acids	Acetic acid D	64-19-7	C2H4O2	60.1	1450.1	1185.707	1.05031
3	Acids	Propanoic acid	1979/9/4	C3H6O2	74.1	1516.8	1528.461	1.11425
4	Alcohols	1- butanol D	71-36-3	C4H10O	74.1	1154.2	575.85	1.38266
5	Alcohols	1- butanol M	71-36-3	C4H10O	74.1	1156.5	580.121	1.1799
6	Alcohols	1-propanol, 2-methyl M	78-83-1	C4H10O	74.1	1108.2	496.946	1.16884
7	Alcohols	1-propanol, 2-methyl D	78-83-1	C4H10O	74.1	1104.2	490.524	1.36716
8	Alcohols	1-butanol, 3-methyl D	123-51-3	C5H12O	88.1	1220.3	686.397	1.49531
9	Alcohols	1-butanol, 3-methyl M	123-51-3	C5H12O	88.1	1224.3	691.748	1.24588
10	Alcohols	Linalool oxide	60047-17-8	C10H18O2	170.3	1482.8	1342.965	1.25618
11	Alcohols	1-octen-3-ol	3391-86-4	C8H16O	128.2	1475.4	1305.829	1.15054
12	Alcohols	2,3-butandiol	513-85-9	C4H10O2	90.1	1553.7	1758.932	1.36428
13	Alcohols	1-penten-3-ol	616-25-1	C5H10O	86.1	1167.9	601.587	0.94199
14	Alcohols	1-propanol D	71-23-8	C3H8O	60.1	1051	423.802	1.26505
15	Alcohols	1-propanol M	71-23-8	C3H8O	60.1	1055.4	428.818	1.11354
16	Alcohols	Ethanol D	64-17-5	C2H6O	46.1	954.8	335.818	1.13166
17	Alcohols	Ethanol M	64-17-5	C2H6O	46.1	968.5	345.464	1.04276
18	Aldehydes	2-furaldehyde D	1998/1/1	C5H4O2	96.1	1446.1	1167.982	1.33664
19	Aldehydes	2-furaldehyde M	1998/1/1	C5H4O2	96.1	1445.3	1164.437	1.0854
20	Aldehydes	1-non-anal	124-19-6	C9H18O	142.2	1401.3	984.827	1.47559
21	Aldehydes	1-hexanal D	66-25-1	C6H12O	100.2	1098.2	481.298	1.5568
22	Aldehydes	1-hexanal M	Jan-25	C6H12O	100.2	1098.7	481.961	1.26113
23	Aldehydes	5-methyl-2-furancarboxaldehyde	620-02-0	C6H6O2	110.1	1527.5	1591.874	1.12878
24	Aldehydes	2-hexenal	505-57-7	C6H10O	98.1	1228.4	697.168	1.18142
25	Aldehydes	(E)-2-pentenal D	1576-87-0	C5H8O	84.1	1144.4	558.043	1.36065
26	Aldehydes	(E)-2-pentenal M	1576-87-0	C5H8O	84.1	1144.4	557.988	1.10449
27	Aldehydes	(E)-2-heptenal	18829-55-5	C7H12O	112.2	1328	846.551	1.25541
28	Aldehydes	Propanal D	123-38-6	C3H6O	58.1	794	263.153	1.14609
29	Aldehydes	Propanal M	123-38-6	C3H6O	58.1	799	264.886	1.0561
30	Aldehydes	Heptanal D	111-71-7	C7H14O	114.2	1192	649.888	1.69216
31	Aldehydes	Heptaldehyde M	111-71-7	C7H14O	114.2	1190.7	647.296	1.33248
32	Aldehydes	Benzaldehyde	100-52-7	C7H6O	106.1	1556.8	1779.506	1.15943
33	Aldehydes	n-pentanal D	110-62-3	C5H10O	86.1	1000.3	369.993	1.42089
34	Aldehydes	n-pentanal M	110-62-3	C5H10O	86.1	1001.3	370.906	1.1893
35	Aldehydes	2-methyl butanal D	96-17-3	C5H10O	86.1	934	321.657	1.40141
36	Aldehydes	2-methyl butanal M	96-17-3	C5H10O	86.1	929.9	318.921	1.16116
37	Aldehydes	2-methyl propanal	78-84-2	C4H8O	72.1	812.5	269.664	1.28133
38	Esters	Butyl acetate M	123-86-4	C6H12O2	116.2	1074.1	450.921	1.23139
39	Esters	Butyl acetate D	123-86-4	C6H12O2	116.2	1081.2	459.484	1.61659
40	Esters	Acetic acid ethyl ester D	141-78-6	C4H8O2	88.1	911.8	307.258	1.33119
41	Esters	Ethyl pentanoate	539-82-2	C7H14O2	130.2	1155.6	578.425	1.264
42	Esters	Acetic acid ethyl ester M	141-78-6	C4H8O2	88.1	913	307.977	1.1038
43	Esters	Methyl 2-methylbutanoate	868-57-5	C6H12O2	116.2	1011.7	381.394	1.1893
44	Heterocycles	2,6-dimethyl pyrazine D	108-50-9	C6H8N2	108.1	1361.5	904.746	1.53802
45	Heterocycles	2,6-dimethyl pyrazine M	108-50-9	C6H8N2	108.1	1361.9	905.367	1.14188
46	Heterocycles	2,3-diethyl-5-methylpyrazine	18138-04-0	C9H14N2	150.2	1502.1	1445.09	1.28297
47	Ketones	1 -hydroxy-2-propanone D	116-09-6	C3H6O2	74.1	1308.9	815.027	1.22799
48	Ketones	1-hydroxy-2-propanone M	116-09-6	C3H6O2	74.1	1315.5	825.704	1.05512
49	Ketones	3-hydroxybutan-2-one D	513-86-0	C4H8O2	88.1	1295.2	793.156	1.32892
50	Ketones	3-hydroxy-2-butanone M	513-86-0	C4H8O2	88.1	1322.7	837.623	1.06657
51	Ketones	Heptan-2-one	110-43-0	C7H14O	114.2	1185.1	635.805	1.26279
52	Ketones	Cyclohexanone M	108-94-1	C6H10O	98.1	1306	810.258	1.14692
53	Ketones	Cyclohexanone D	108-94-1	C6H10O	98.1	1307.4	812.511	1.4572
54	Ketones	1-penten-3-one	1629-58-9	C5H8O	84.1	1037.1	408.298	1.31051
55	Ketones	4-methyl-2-pentanone	108-10-1	C6H12O	100.2	1027.4	397.81	1.17847
56	Ketones	2-butanone D	78-93-3	C4H8O	72.1	922.3	313.966	1.24686
57	Ketones	2-butanone M	78-93-3	C4H8O	72.1	918.1	311.223	1.06552
58	Ketones	2-propanone	67-64-1	C3H6O	58.1	822.8	273.32	1.11246
59	Organosulfur compounds	Allyl isothiocyanate	1957/6/7	C4H5NS	99.2	1380	938.54	1.09029
60	Organosulfur compounds	Diallyl disulfide	2179-57-9	C6H10S2	146.3	1483.2	1345.214	1.19955

The substance suffixes M and D represent monomers and dimers of the same substance, respectively.

#### 3.2.3 GC–IMS profile analysis of VOCs in four samples

**FIGURE 3:**
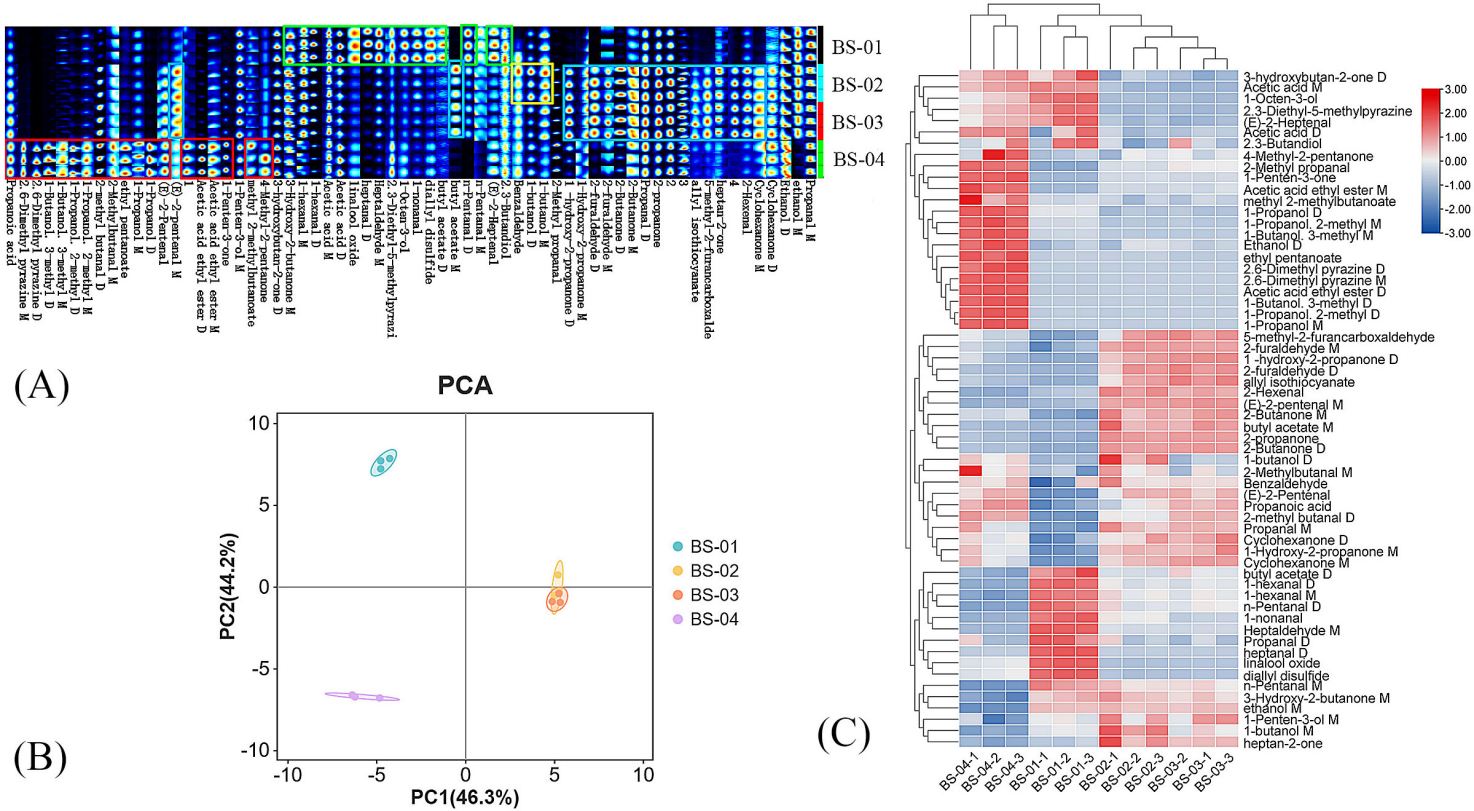
**(A)** Fingerprint of VOCs in four samples; **(B)** PCA score plot of VOCs in four samples; **(C)** cluster analysis of VOCs in four samples.

Figure 3A shows the fingerprint of PRA processed using different methods. Each row represents all of the selected signal peaks from a sample, and each column represents the signal peaks of the same volatile organic compound in different samples. The red boxes indicate the following: propanoic acid; 2,6-dimethyl pyrazine M; 2,6-dimethyl pyrazine D; 1-butanol, 3-methyl D; 1-butanol, 3-methyl M; 1-propanol, 2-methyl D; 1-propanol, 2-methyl M; 2-methyl butanal D; 2-methyl butanal M; ethyl pentanoate; 1-propanol M; 1-propanol D; (E)-2-pentenal; acetic acid ethyl ester D; acetic acid ethyl ester M; 1-penten-3-one; methyl 2-methyl butanoate; 4-methyl-2-pentanone. The contents of BS-04 are relatively high. The green boxes indicate the following: 3-hydroxy-2-butanone M; 1-hexanal M; 1-hexanal D; acetic acid M; acetic acid D; linalool oxide; heptanal D; heptaldehyde M; 2,3-diethyl-5-methylpyrazine; 1-octen-3-ol; 1-non-anal; diallyl disulfide; butyl acetate D; n-pentanal D; (E)-2-heptenal; 2,3-butandiol. The contents of BS-01 are relatively high. As shown in the yellow box, benzaldehyde, 1-butanol D, and 1-butanol M are present in higher amounts in BS-02. As shown in the blue boxes, (E)-2-Primary M, butyl acetate M, 1-hydroxy-2-proline D, 1-hydroxy-2-proline M, 2-furaldehyde D, 2-furaldehyde M, 2-butanone D, 2-butanone M, propanal D, 2-propanone, allyl isothiocyanate, 5-methyl-2-furancarboxylic acid, heptan-2-one, 2-hexenal, and cyclohexanone M are present in higher amounts in BS-02 and BS-03.

#### 3.2.4 PCA of VOCs

In PCA, components with a cumulative contribution rate greater than 60% are typically considered to capture most of the information of a sample, and samples with similar VOCs appear closer together in the PCA. Figure 3B shows a two-dimensional score plot based on two principal components. The contribution rates of the first and second principal components are 46.3% and 44.2%, respectively, and the cumulative contribution rate of the principal components is 90.5%, meaning that they represent the majority of the original variable information for VOCs and reflect the main characteristics of the sample well. The close proximity of each group indicates that their differences are minimal and the sample reproducibility is good. BS-02 and BS-03 are relatively close in Figure 3B, while they are far away from BS-01 and BS-04, indicating that stir-baking and stir-baking with bran result in more similar changes in the VOCs of PRA, and the correlation is strong.

#### 3.2.5 CA

The cluster analysis results are shown in Figure 3C, where the color depth indicates the concentration of the VOC, with higher contents corresponding to darker colors (red indicates upregulation, and blue indicates downregulation). The hierarchical dendogram of the cluster analysis shows that BS-02 and BS-03 have obvious clustering, while BS-04 differs significantly from the other three groups. This is consistent with the PCA results presented in the text above. In the BS-01 group, the contents of N-pentanal m diallyl disulfide, linalool oxide, heptanal D, propanal D, heptaldehyde M, 1-non-anal, n-pentanal D, 1-hexanal M, 1-hexanal D, and butyl acetate D were significantly higher than those in the other groups, and the contents of cyclohexanone M, 1-hydroxy-2-propanone M, cyclohexanone D, propanal M, 2-methyl butanal D, propanoic acid, (E)-2-pentenal, 2-butanone D, 2-propanone, 2-butanone M, allyl isothiocyanate, 1-hydroxy-2-propanone D, 5-methyl-2-furancarboxaldehyde, acetic acid ethyl ester M, 1-penten-3-one, and 2-methyl propanal were lower than those in the other groups. Heptan-2-one, 1-butanol M, and 1-butanol D were significantly higher in the BS-02 group than in the other groups, and cyclohexanone M and 1-hydroxy-2-propanone M were significantly higher in the BS-03 group than in the other groups. In the BS-04 group, the contents of 1-propanol M; 1-propanol 2-methyl D; 1-butanol 3-methyl D; acetic acid ethyl ester D; 2,6-dimethyl pyrazine M; 2,6-dimethyl pyrazine D; ethyl pentanoate; ethanol D; 1-butanol 3-methyl M; 1-propanol 2-methyl M; 1-propanol D; methyl 2-methylbutanoate; acetic acid ethyl ester M; 1-penten-3-one; and 2-methyl propanal were significantly higher than those in the other groups, but heptan-2-one; 1-butanol M; 1-penten-3-ol M; ethanol M; 3-hydroxy-2-butanone M; n-pentanal M; heptaldehyde M; 1-non-anal; n-pentanal D; 1-hexanal M; 1-hexanal D; butyl acetate D; (E)-2-pentenal M; and 2-hexenal were lower than those in the other groups.

#### 3.2.6 PLS-DA

**FIGURE 4:**
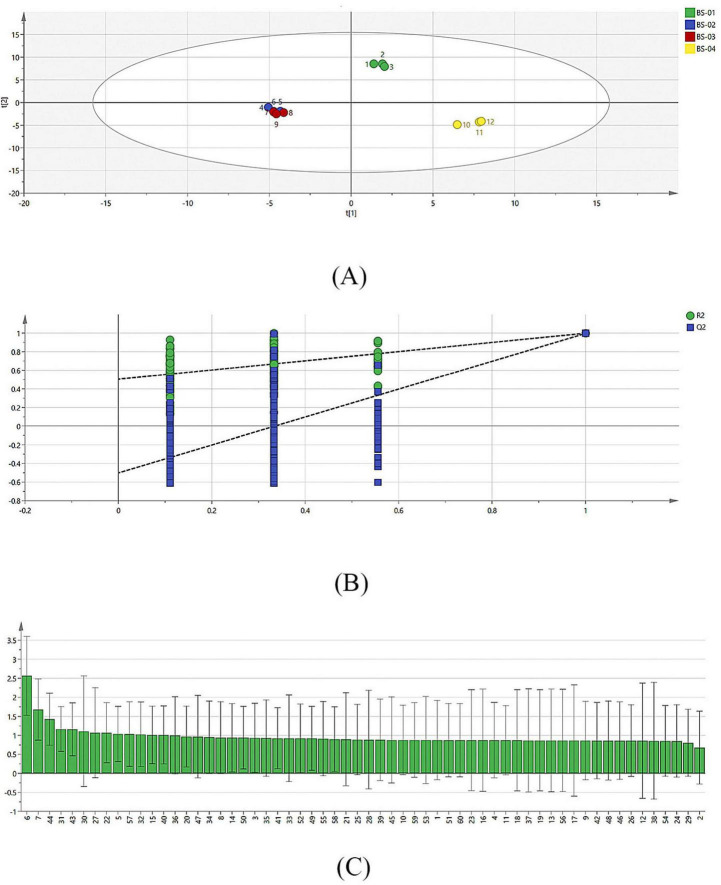
PLS-DA results of VOCs in four samples. **(A)** PLS-DA models for VOCs in four samples; **(B)** significance diagnostic: 200 cross-validations were performed using displacement test analysis; **(C)** the VIP values of VOCs which VIP > 1.

Partial least squares regression analysis was conducted on the relative content data for the VOCs of PRA samples processed using different methods (Figure 4A). A PLS-DA model was established to examine its effectiveness in modeling the data. In total, 200 cross-validations were performed using displacement test analysis, as shown in Figure 4B. Through the PLS-DA, each volatile compound was assigned a VIP value, which is the variable importance projection. The VIP value can evaluate the strength and explanatory power of each variable’s impact on classification and discrimination. In the discrimination process, it is generally believed that a VIP value greater than 1 indicates that the variable has an important role. Therefore, when selecting key VOCs, compounds with VIP values greater than 1 are typically considered the key VOCs for a group of samples. As shown in Figure 4C, the VIP values of 13 VOCs are greater than 1, mainly including 1-butanol D, 1-butanol M, 2-methyl butanal M, propanoic acid, 2-methyl butanal D, 2,3-butandiol, heptan-2-one, allyl isothiocyanate, 1-non-anal, propanal D, 5-methyl-2-furancarboxaldehyde, butyl acetate D, and 4-methyl-2-pentanone. This indicates that the VOCs of PRA processed using different methods differ significantly and are key indicators of VOC differences and that there are many differential biomarkers of VOCs.

### 3.3 Heracles NEO ultra-fast gas phase electronic nose analysis

#### 3.3.1 Gas chromatogram analysis

**FIGURE 5:**
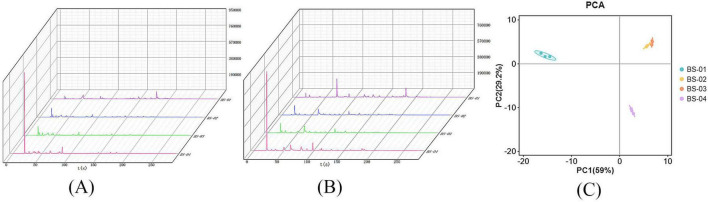
**(A)** MXT-5 gas chromatography results of VOCs in four samples; **(B)** MXT-1701 gas chromatography results of VOCs in four samples; **(C)** PCA of VOCs in four samples.

The Heracles NEO ultra-fast gas phase electronic nose contains two ionization detectors: an MXT-5 low-polarity chromatography column and an MXT-1701 medium-polarity chromatography column. To more accurately compare the differences between samples, we analyzed the detection results from both chromatography columns and overlaid the original gas chromatograms of all of the PRA samples. The results are presented in Figures 5A, B. Figures 5A, B displays data from multiple samples, each represented by a distinct color. The gas chromatography overlay visually demonstrates that the detection results of the two chromatography columns are generally similar, but there are differences in the retention time and peak area among the four types of PRA samples. The spectrum shows that the chromatographic peak of the BS-01 sample, represented by purple, is generally low, between 0 and 50 s, with a characteristic peak around 20 s. An analysis of this peak reveals that the chromatographic peak of the BS-04 sample, represented by red, is significantly higher than the peaks of the other three PRA samples. The peak height between 100 and 300 s is relatively low, and the purple BS-01 sample has a characteristic peak. An analysis of the original spectra reveals that the main differences among the four samples of PRA are changes in peak height, reflecting differences in VOCs. To further verify the differences between sample groups, we first used PCA statistics to identify odor differences between sample groups and determine differential chromatographic peaks. Then, we qualitatively analyzed specific chromatographic peaks through database retrieval, thus accurately and effectively identifying differences in the VOCs of PRA processed using different methods.

#### 3.3.2 PCA

Principal component analysis was performed on the VOC data for four PRA samples, as shown in Figure 5C. The horizontal and vertical axes represent the contribution rates of the first principal component (PC1) and second principal component (PC2), respectively. In the PCA model, the contribution rate of the first principal component (PC1) is 59%, the contribution rate of the second principal component (PC2) is 29.2%, and the cumulative contribution rate of PC1 and PC2 reaches 88.2%, meaning that it can well reflect the actual situation of the sample. The principal component analysis graph shows that, the smaller the distance between samples, the smaller the sample difference, and the greater the distance, the larger the sample difference. As seen in Figure 5C, the position distribution of BS-02 and BS-03 is the closest, and the overall odor difference between the two groups of samples is relatively small. The distance between BS-01 and BS-04 is the greatest, indicating the greatest odor difference between the BS-01 and BS-04 samples. This is consistent with the principal component analysis results for GC-IMS, verifying the results mentioned above.

#### 3.3.3 Qualitative characterization of compounds

Chromatographic peak analysis was performed using the AroChemBase database to identify compounds with significant differences. Table 2 lists potential compounds based on the qualitative analysis, and the entire compound library was queried using a qualitative search. The threshold size in Table 2 indicates the odor strength. The unit of the threshold is mg/m3 in air and mg/kg in water. Two compounds with the same molecular weight have lower odor thresholds in the same medium, indicating stronger odors. For substances with different contents and thresholds, the odor activity value (OAV) is used. The OAV is the ratio of the sample’s individual substance concentration to its threshold concentration. A high ratio indicates a large OAV and significant odor contribution. A low ratio indicates a small OAV and minimal odor contribution. By knowing each compound’s content in the sample and combining it with the odor threshold, the odor activity value (or odor contribution value) can be calculated. This value guides odor adjustment and has directional significance for odor tracing.

**TABLE 2:** Differential chromatographic peak qualitative results and odor descriptions.

No	Compounds	CAS	RI (RT-5)	RI (RT-1701)	Odor description	Odor threshold
1	Hexane	110-54-3	593	600	Alkane; etheral; gasoline; kerosene	1.69e + 2 (air)
2	Diisopropyl ether	108-20-3	605	611	Camphor; etheral; pungent; sharp; sweet	0.07 (air)
3	Methylcyclopentane	96-37-7	623	643	Gasoline	5.80 (air)
4	Tetrahydrofuran	109-99-9	633	696	Etheral; fruity	1.8e + 1 (air)
5	n-butanol	71-36-3	651	789	Alcoholic; amyl alcohol; banana; cheese; fermented; fruity; fusel; harsh; medicinal; oil; rancid; strong; sweet	1.12 (air)
6	Thiophene	110-02-1	671	745	Alliaceous; aromatic; garlic; sulfurous	1.90 (air)
7	Methyl glycolate	96-35-5	677	–	–	–
8	Pentan-2-ol	6032-29-7	688	814	Alcoholic; etheral; fermented; fruity; fusel; green; green (mild); mild; nutty; oil; plastic; pungent; raspberry; sweet	1.00 (air)
9	Propyl acetate	109-60-4	712	–	Caramelized; celery; fermented; fruity; fusel; ketonic; mild; pear; pleasant; raspberry; solvent; sweet	7.0e + 1 (air)
10	2-methyl-1-butanol	137-32-6	740	857	Alcoholic; balsamic; banana; butter; fusel; iodoform; malty; oil; onion (ripe); sweet; vinous; winey	0.14 (air)
11	Hexanal	66-25-1	803	894	Acorn; aldehydic; fatty; fishy; fresh; fruity; grassy; green; herbaceous; leafy; sharp; strong; sweaty; tallowy; vinous	0.04 (air)
12	Butyl acetate	123-86-4	812	–	Banana; bitter; etheral; fruity; green; pear; pineapple; pleasant; solvent; strong; sweaty; sweet	0.70 (air)
13	1-pentanol, 2,3-dimethyl-	10143-23-4	818	932	–	1.22 (water)
14	Ethyl trans-2-butenoate	623-70-1	830	921	Alliaceous; chemical; pungent; rum; sweet	–
15	Furfural	1998/1/1	837	985	Almond; baked; benzaldehyde; bread; fragrant; sweet; woody	0.98 (air)
16	2-methyl-2-cyclopenten-1-one	1120-73-6	918	1028	–	–
17	Decane	124-18-5	999	993	Alkane; fruity; fusel; sweet	1.13e + 1 (air)
18	1,3,5-trimethylbenzene	108-67-8	1014	1012	Aromatic; herbaceous	1.20 (air)
19	Alpha-phellandrene	99-83-2	1022	1021	Citrus; green; minty; spicy; terpenic; turpentine; woody	3.40 (air)
20	Decane, 5-methyl-	13151-35-4	1058	1055	–	–
21	n-non-anal	124-19-6	1123	1199	Aldehydic; chlorine; citrus; fatty; floral; fresh; fruity; gaseous; gravy; green; lavender; melon; orange; orange peel; orris; peely; pungent (slightly); rose; soapy; sweet; tallowy; waxy	0.01 (air)
22	Heptanoic acid	111-14-8	1151	1290	Cheese; faint; fatty; rancid; sour; sour-sweat (sour)	0.17 (air)
23	3-decanol	1565-81-7	1196	1308	Fatty; floral; mushroom; musty; orange	0.24 (water)
24	6-decenal	147159-48-6	1204	1314	Cucumber; green	3.e-3 (air)
25	L-carvone	2244-16-8	1266	1397	Bread; caraway; herbaceous; minty; spicy	0.03 (air)

The average peak areas of the PRA samples at different retention times (MXT-5 or MXT-1701 was selected based on the peak state) are listed in Table 3. Because the detector is both an FID detector and a mass detector, the average peak area is higher when the content of the same substance is high.

**TABLE 3:** Average peak area of differential chromatographic peaks.

No	Compounds	CAS	Average peak area			
			BS-01	BS-02	BS-03	BS-04
1	Hexane	110-54-3	10478	45925	49219	401235
2	Diisopropyl ether	108-20-3	2471	18574	19631	5836
3	Methylcyclopentane	96-37-7	147	13546	20665	23375
4	Tetrahydrofuran	109-99-9	0	6779	7208	4633
5	n-butanol	71-36-3	1057	44371	79665	23659
6	Thiophene	110-02-1	2184	10265	15755	31487
7	Methyl glycolate	96-35-5	0	23193	31660	23776
8	Pentan-2-ol	6032-29-7	110844	3563	3329	3497
9	Propyl acetate	109-60-4	0	10916	10773	13216
10	2-methyl-1-butanol	137-32-6	0	0	0	49936
11	Hexanal	66-25-1	25648	12800	12347	23409
12	Butyl acetate	123-86-4	6819	3673	3736	2579
13	1-pentanol, 2,3-dimethyl-	10143-23-4	16584	682	682	70211
14	Ethyl trans-2-butenoate	623-70-1	0	8409	8438	0
15	Furfural	1998/1/1	493	27703	30406	3510
16	2-methyl-2-cyclopenten-1-one	1120-73-6	0	11386	12024	1061
17	Decane	124-18-5	9389	6013	5594	5328
18	1,3,5-trimethylbenzene	108-67-8	5713	4875	4638	3322
19	Alpha-phellandrene	99-83-2	13550	8594	8493	7089
20	Decane, 5-methyl-	13151-35-4	28165	16547	17171	14855
21	n-non-anal	124-19-6	540	530	551	7949
22	Heptanoic acid	111-14-8	13848	8296	7569	15973
23	3-Decanol	1565-81-7	81232	10859	10996	17770
24	6-decenal	147159-48-6	26806	8100	8582	10795
25	L-carvone	2244-16-8	14108	6139	6437	7874

As shown in Table 2, a total of 25 odor components were identified, and it can be seen that BS-04 contains hexane, methylcyclopentane, thiophene, propyl acetate, 2-nethyl-1-butanol, 1-pentanol, 2,3- dimethyl-, n-non-anal, and heptanoic acid. The contents of each compound were the highest in this sample, and hexane was significantly higher in this sample, while the contents of ethyl trans-2-butanoate, furfural, and 2-methyl-2-cyclopenten-1-one were lower in the other three samples. The compound with the highest content in PRA is hexane, with the BS-04 sample having the highest content. In BS-01, the contents of pentan-2-ol, hexanal, butyl acetate, 3-decanol, 6-decenal, L-carvone, decane, 1,3,5-trimethylbenzene, alpha-phellandrene, and 5-methyldecane were higher than those in the other samples. The contents of diisopropyl ether, n-butanol, methyl glycolate, furfural, and 2-methyl-2-cyclopenten-1-one in BS-03 were higher than those in the other samples, while the contents of the other compounds were relatively low. BS-02 did not contain any components that were significantly higher than those in the other PRA samples but contained components similar to those in BS-03. The contents of diisopropyl ether, n-butanol, furfural, and 2-methyl-2-cyclopenten-1-one in BS-02 were higher than those in BS-01 and BS-04 but slightly lower than those in BS-03, and the contents of other compounds were generally lower than those in the other samples. The data in the table show that the chemical compositions of the VOCs in PRA are relatively similar, but there are significant differences among the different processing methods, indicating that there are differences in quality.

## 4 Discussion

This study utilized both GC-IMS and a Heracles NEO ultra-fast gas phase electronic nose to analyze the differences in the VOCs of PRA under various processing methods. A total of 85 VOCs were detected, i.e., 24 aldehydes, 19 alcohols, 14 ketones, 11 esters, 5 heterocycles, 4 organic acids, 4 alkanes, 1 aromatic hydrocarbon, 1 sulfide, 1 ether, and 1 terpene.

By conducting PCA on the 60 VOCs detected via GC-IMS, it was found that the differences in VOCs between BS-02 and BS-03 were relatively small, while BS-04 showed the largest difference in VOCs compared to the other three groups. The changes in VOCs were significant, consistent with the CA results and validated by the PCA results for the electronic nose. The combination of the electronic nose and GC-IMS maximally preserves the relevant information of VOCs in PRA (24, 25), and their results can be verified. Multivariate statistical analysis was applied to the data obtained to rapidly differentiate PRA samples processed using different methods (26).

Using GC-IMS and the electronic nose, a comparative analysis of the VOCs in PRA processed with different methods was performed. We found that the content of linalool oxide, which possesses anticonvulsant effects, was high in BS-01 (27), while it was relatively low in the other three groups, indicating that the three processing methods reduced the linalool oxide content, thereby diminishing the anticonvulsant effects of Paeoniae Radix Alba. Propanoic acid has been shown to reduce intestinal inflammation, and its content in BS-04 was higher than that in the other three groups (28, 29). Therefore, while the anticonvulsant effects of PRA stir-baked with wine are not as potent as those of prepared slices of Paeoniae Radix Alba, the ability of the samples processed by stir-baking with wine to reduce intestinal inflammation is more robust than that of the other three methods. According to GC-IMS and the PCA of the electronic nose, there is little difference in VOCs between BS-02 and BS-03, the contents of furfural components with anti-allergic, antioxidant, and anti-hypoxic pharmacological activities in both groups of PRA were higher than in the other two groups (30, 31), indicating that these activities of PRA (stir-baked or stir-baked with bran) were improved to some extent. The results of this study contribute to an understanding of the VOCs and differences in PRA samples subjected to different processing methods, aiding in the selection of the most appropriate processing methods and product development applications.

The combination of two technologies enables the determination of the relative content of VOCs of PRA under different processing methods, providing a fast, accurate, and feasible analysis strategy. This provides new insights into the quality evaluation of different processed products of PRA and offers technical support for the future clinical application of PRA and the development of functional foods. However, our research still has shortcomings. The mechanisms by which different processing methods have an impact are not yet clear. For example, why wine increases the compound propanoic acid, Is it due to microbial fermentation, heat treatment, Maillard reaction, or other reasons? In addition, besides the processing methods mentioned in our study, the impact of other processing methods is also unknown. In addition, our sample size is not large enough. In the future, we will further investigate and provide a more systematic and comprehensive elucidation of the effects and mechanisms of different processing methods on *Paeonia lactiflora*.

## 5 Conclusion

Gas chromatography–ion mobility spectrometry and E-nose technologies, coupled with chemometric methods, can effectively distinguish the VOCs of PRA under different processing methods. This research showed that different processing methods have a significant impact on the VOCs of PRA. PRA (prepared slices), which had not undergone special processing, exhibited high levels of linalool oxide in its VOCs. This compound may possess anticonvulsant effects, highlighting the unique pharmacological advantages of PRA (prepared slices). During stir-baking and stir-baking with bran, the VOCs with potential pharmacological activities, such as anti-allergy, antioxidant, and anti-hypoxia effects, increased, indicating that these two processing methods have enhance the potential of PRA in the treatment of various diseases. The content of propanoic acid, which may have the effect of reducing intestinal inflammation, in PRA (stir-baked with wine) was significantly higher than that in the other processed PRA products. These findings provide a scientific basis for the development of functional foods from PRA and the application of clinical treatments in the future.

## Data Availability

The original contributions presented in this study are included in this article/supplementary material, further inquiries can be directed to the corresponding author.
